# Educational Aspirations and Expectations of Adolescents in Rural China: Determinants, Mental Health, and Academic Outcomes

**DOI:** 10.3390/ijerph182111524

**Published:** 2021-11-02

**Authors:** Xiaodi Chen, Therese Hesketh

**Affiliations:** 1 Institute for Global Health, University College London, London WC1N1EH, UK; xiaodi.chen.17@ucl.ac.uk; 2 Center for Global Health, Zhejiang University School of Medicine, Hangzhou 310058, China

**Keywords:** educational aspirations, educational expectations, mental health, academic outcomes, adolescents, rural China, left behind children, cross-sectional studies

## Abstract

Educational aspirations and expectations of adolescents have implications for educational and psychological outcomes. This study aimed to determine factors associated with educational aspiration–expectation discrepancies and to examine the associations between the discrepancies and psychological and academic outcomes in rural left-behind children and non-left-behind children aged 14–16 in mainland China. Cross-sectional data from a self-report survey were collected in 2020 among 606 rural students (mean age = 14.85 years) in two public middle schools in Songzi county, Hubei Province. Participants filled in questionnaires measuring their socio-demographic information, educational aspirations and expectations, academic performance, parental and friends’ aspirations, academic self-perception, academic self-regulation, depression, and self-esteem. Results showed that more than half of the participants reported that they felt they were not likely to attain the level of education to which they aspired. Parental migration, academic performance, mother’s educational aspirations for children, and close friends’ educational aspirations were the main factors associated with students’ educational aspiration–expectation discrepancies. Both left-behind children and non-left-behind children whose aspirations exceed expectations were more likely to report lower self-esteem, higher depression, lower academic self-perception, and poorer self-regulation than those without a discrepancy. These findings have implications for families, schools, and policymakers through informing the development of interventions that target positive development in rural youth.

## 1. Introduction

Educational aspirations and expectations are individuals’ beliefs regarding educational future plans. Educational aspirations are idealistic values that reflect the educational attainment that one hopes and desires to achieve [1]. Educational expectations are concrete values determined by perceived realities that are faced by an individual, which usually consider personal abilities and other constraints [2]. Thus, there is the potential for discrepancies between the aspired and expected educational attainment. During adolescence, youth begin to think about future opportunities and goals [3]. Previous studies have demonstrated that educational aspiration–expectation discrepancies are widespread among adolescents. For example, Kirk et al. [4] found in a sample of 207 8th-grade students in the United States that 47% reported at least a one-level gap between their educational aspirations (e.g., obtaining a master’s degree) and expectations (e.g., obtaining a bachelor’s degree).

The Chinese strongly value education and adolescents usually have high educational goals [5] with rural adolescents seeing education as a route out of poverty [6]. Educational attainment remains an important determinant of rewarding jobs and high incomes [7]. Rural adolescents are often especially motivated in pursuing higher education. However, they often face barriers to quality education compared with their urban counterparts [8]. For example, around 38% of rural students nationwide were unable to complete 9 years of compulsory education [9] and adolescents from poor rural areas were 7 and 11 times less likely to go to any universities and elite universities than urban adolescents [10]. The disparity suggests that rural adolescents expect to encounter more barriers (e.g., parenting and educational resources) to pursuing education [8].

In China, rural-to-urban migration has occurred since the late 1970s. Many migrants flow from inland rural areas to eastern coastal cities. China had an estimated 174 million rural-to-urban migrants in 2019 [11]. Most rural migrant workers are engaged in unskilled and low-income jobs in urban settings. Because of the long working hours, the high living costs in urban settings, and restrictions associated with the household registration (hukou) system [12], many migrant workers leave their children in their hometown while they go to the cities to work. Rural left-behind children (LBC) refer to children under 18 who are left behind in their rural hometowns, while one or both of their parents have migrated to a different location for work for at least six months [13]. According to the 2015 1% National Population Sample Survey, there were approximately 40.5 million rural LBC in China, accounting for 29.4% of all rural children. Of these, 51% lived with one parent (usually mothers), 39% lived with their grandparents or other relatives, 3% lived alone, and 7% lived with other children [9]. In the present study, adolescents left behind for at least six months by both parents are referred to as LBCBs, left by one parent as LBCOs, and living with both parents as non-LBC or NLBC.

Parental migration may be associated with educational aspiration–expectation discrepancies among rural adolescents. The financial remittances earned from parental migration may have a beneficial effect on children’s education [14]. In addition, there are social remittances which relate to the parents’ urban lifestyle and access to information about educational and occupational opportunities, which may broaden children’s horizons [15]. However, parental absence leads to the loss of parental attention and supervision [16], which may have detrimental effects on children’s development. LBC have been found to have worse academic performance [17], higher dropout rates [15], and more mental health problems than NLBC [18,19]. Moreover, in the absence of parents, LBC are often required to undertake more household chores, which may restrict their school attendance and homework time [20,21]. These may lead to lower educational expectations of LBC.

In addition to parental migration, there are other factors have been shown to account for the discrepancies, such as parental influence on children’s educational beliefs [22,23]. Adolescents often have similar outlooks on the world to their parents and use this information in considering what they expect for themselves [24]. In the Chinese context, many parents in rural areas recognise education as the means for upward mobility [25] and subsequently set high educational aspirations for their children, which may in turn influence children’s aspirations. However, educational resources are more limited in rural areas [26], and lack of parental support (especially for LBC) makes it more difficult to achieve good educational outcomes [27].

Researchers focus on educational aspiration–expectation discrepancy because of its adverse effects on mental health. According to the “self-discrepancy theory” [28], people have an ideal self (the attributes a person aspires to possess) and an actual self (the attributes the person actually possesses). The discrepancy between the two self-representations is associated with poor mental health outcomes, such as depression [29,30].

Educational aspirations and expectations are examples of the “ideal (or hoped-for) self” and “actual self (or expected)” within the academic domain, which suggests that students with academic discrepancies may be at risk of developing low well-being. Rutherford et al. [31] has examined the educational aspiration–expectation discrepancies among a national representative sample of middle and high school students in the US (*n* = 1115). The discrepancies are associated with lower emotional well-being of high school students. Additional empirical evidence from the US has also shown that adolescents who aspire to attain more than they expect to attain are more likely to report depression [32], anxiety [33], and behavioural and emotional difficulties [33]. Some scholars have noted that dreaming big but developing a mindset characterised by fear of unsuccess is related to negative emotions even if people achieve their goals [34].

The potential negative effects of educational aspiration–expectation discrepancies are also notable in academic domain. From the perspective of possible-selves, educational aspirations and expectations are school-related selves which adolescents believe they might become in the future and therefore guide their goal-directed behaviours [35]. Those with matched possible selves have been shown to be better at regulating their progress in achieving their goals [36]. Thus, Kirk et al. [4] noted that the US middle school students whose educational aspirations exceed their expectations report less self-regulating behaviours in school. In addition, aspiring high while expecting low has also been shown to have a negative effect on adolescents’ perceptions of their academic abilities [4].

The current study explored educational aspirations, expectations, and their discrepancies in LBC and NLBC in rural China. While research conducted in Western societies has evidenced the important role of educational aspiration–expectation discrepancies in adolescents’ mental health and academic outcomes, this has not been studied in China. To the best of our knowledge, this is the first study to address this topic for rural adolescents. The study had three aims: (1) to investigate educational aspirations, expectations, and the aspiration–expectation discrepancies in rural LBCOs, LBCBs and NLBC; (2) to explore the factors associated with educational aspiration–expectation discrepancies; (3) to explore the associations between the educational aspiration–expectation discrepancies and the mental health and academic outcomes.

## 2. Materials and Methods

### 2.1. Setting and Participants

The study was conducted in Songzi, a middle-SES rural county in Hubei Province, Central China. Songzi has 17 townships and participants were sampled from Nanhai town, which has 2 middle schools and 21 villages. The participants comprised students enrolled in Grade 9 of the two middle schools in 2019. This is the last year of compulsory education when students have to think about their educational future. According to records from the two schools, only around 60% of the students in Nanhai continue to high school, while the rest enter vocational and technical schools or start work immediately after middle school graduation.

### 2.2. Procedure

Following the receipt of approval from the university ethics board, the approval of the headteacher at the two public middle schools was obtained. Data were collected in the schools in June 2020. Students and parents (or caregivers) were required to provide written consent participation. The self-completion survey took approximately 40 min to complete and was administered in the classroom during school hours under the supervision of a researcher, who was available to answer questions throughout the time period. The survey responses were anonymous and confidential.

### 2.3. Measures

Socio-demographic information included age, sex, only child or not, parents’ education levels, parents’ marital status, the primary caregiver, and left-behind characteristics.

Educational aspirations and expectations were measured with two questions: “What is the highest level of education that you … wish to achieve?” and “… think that you will achieve?”. The responses were made on a 6-point scale (1 = completion of middle school; 2 = vocational school; 3 = high school; 4 = bachelor’s degree; 5 = master’s degree; 6 = doctoral degree). Items were based on similar measures used in prior studies of educational aspirations and expectation in Chinese children and adolescents [37].

Academic performance: Participants were asked to indicate “Which of the following best describes your academic performance in class in comparison with other students this year?”. The responses were made on a 5-point scale (1= low, ranked lower than 50; 2 = low-middle, ranking 30–50; 3 = middle, ranking 20–30; 4 = upper-middle, ranking 10–20; and 5 = top, ranking top 10). As in most of China, students are informed of their ranking in the class for tests; thus, they can report their academic performance without difficulty. Academic ranking as an indicator of academic performance has been used in prior studies in Chinese samples [38].

Economic status: Participants completed the Family Affluence Scale (FAS II) [39] which includes 4 items that measure universal aspects of economic status (e.g., “How many computers does your family own?”). The responses were made on a 3-point scale (0 = None (No); 1 = Once (One); 2 = More than once (one), except that having a separate bedroom was coded as No (=0) and Yes (=1) rather than scales. Three groups were categorised based on the composite FAS score: FAS low (score = 0–3), FAS medium (score = 4, 5), and FAS high (score = 6, 7). The reliability and validity of the Chinese version of the FAS II were assessed [40]. The Cronbach’s alpha coefficient was 0.45.

Adolescents’ perceived parental aspirations: Participants reported their perception of parents’ educational aspirations for them (“How far do you think your mother and father would like you to go in school?”) on a 6-point scale ranging from 1 = completion of middle school to 6 = doctoral degree. Similar measures were used in previous research [41].

Friends’ aspirations: Participants responded to a question regarding the aspirations of their close friends (“As far as you know, what is the highest level of education that your close friends would like to achieve?”). The responses were made on a 6-point scale from 1 = completion of middle school to 6 = doctoral degree.

Academic self-perception is defined as the perception adolescents have about their academic abilities (e.g., “I can learn new ideas quickly in school”), was assessed using 7 items adapted from the School Attitude Assessment Survey-Revised (SAAS-R) [42]. The responses were made on a 7-point scale from 1 = disagree very strongly to 7 = agree very strongly. The Cronbach’s alpha coefficient was 0.89.

Academic self-regulation, which is the process by which students manage their thoughts and behaviours in order to achieve their academic goals [43], was assessed with 10 items from the SAAS-R [40]. Participants were asked to rate a sample question, such as “I concentrate on my schoolwork”, on a 7-point scale. The Cronbach’s alpha coefficient was 0.93.

Depression: The participants completed the Children’s Depression Inventory—Short Version (CDI-S) [44] which includes 10 items measuring various indicators of youth-specific depressive symptoms (e.g., sadness, pessimism, and self-deprecation). The responses were made on a 3-point scale (0 = the symptom is not present; 1 = mild symptoms; 2 = definite symptoms). The CDI-S has been commonly used as a screening instrument for depressive symptoms in children and adolescents (7–17 years) in China [45]. Based on previous research [46], a cutoff CDI-S score of 4 was accepted for depression risk. The Cronbach’s alpha coefficient was 0.81.

Self-esteem was assessed with 10 items from the Rosenberg Self-esteem Scale (RSES) [47]. The responses were made on a 4-point scale from 1 = “strongly disagree” to 4 = “strongly agree”. A cutoff score of 25 was used to identify adolescents with low self-esteem [48]. The RSES has demonstrated appropriate psychometric properties in prior studies with Chinese youth [49]. The Cronbach’s alpha coefficient was 0.90.

### 2.4. Analysis

First, we generated descriptive statistics on demographic information and educational aspiration–expectation discrepancies. Next, logistic regression was used to examine the predictors of aspiration–expectation discrepancy, which were treated as a dichotomous dependent variable (0 = not having the discrepancies and 1 = having the discrepancies). The Hosmer–Lemeshow goodness-of-fit tests were used to evaluate the degree of fitness of the models, and Cox and Snell’s R^2^ and Nagelkerke’s R^2^ were calculated to examine the amount of variation in the dependent variable explained by the model. Finally, ANCOVA was used to examine the impact of aspiration–expectation discrepancies on the mental health and academic outcomes of LBCB, LBCO, and NLBC, while controlling for the influence of academic performance. Data analyses were performed with IBM SPSS 27.0.

## 3. Results

Table 1 shows the demographic information and left-behind characteristics of the sample. The sample for the present study consisted of 606 adolescents (303 females and 303 males; *M* age = 14.85 years, *SD* = 0.59). Based on their report of parental migration and the time of maternal and paternal migration, adolescents were categorised into three groups: LBCO (*n* = 169, 27.9%), LBCB (*n* = 273, 45.59%), and NLBC (*n* = 164, 27.1%). Significant differences were observed in terms of parental marital status and family economic status: 24.2% of the LBCB parents were divorced, compared with 7.1% of the LBCO parents and 4.9% of the NLBC parents. NLBC reported higher economic status at 20.1%, when compared to 12.1% LBCBs and 14.8% LBCOs. Moreover, there were significant differences between LBCOs and LBCBs. First, most LBCBs were raised by grandparents, and all LBCOs were raised by their non-migrant parent (usually mothers). Second, most LBC reported that their parent(s) had worked away for more than 5 years. LBCBs experienced longer maternal absence than LBCOs. Third, LBCOs had more frequent contact with the migrant parent than LBCBs.

Table 2 shows the binary correlations between the main study variables. Educational expectations of adolescents were positively correlated with parents’ educational level (mother: *r* = 0.13, *p* < 0.01; father: *r* = 0.14; *p* < 0.01). The associations between aspirations and parents’ educational level were non-significant (all *p* > 0.05). Educational aspirations and expectations were positively associated with academic self-perception (aspirations: *r* = 0.26, *p* < 0.01; expectations: *r* = 0.44, *p* < 0.01), academic self-regulation (aspirations: *r* = 0.29, *p* < 0.01; expectations: *r* = 0.50, *p* < 0.01), and self-esteem (aspirations: *r* = 0.20, *p* < 0.01; expectations: *r* = 0.26, *p* < 0.01) and negatively associated with depression (aspirations: *r* = −0.12, *p* < 0.01; expectations: *r* = −0.20, *p* < 0.01).

Table 3 presents the descriptive results of educational aspirations and expectations. The majority within all three groups had high aspirations and expectations. Specifically, more than 50% rural adolescents aspired to attain a master’s or doctoral degree and more than 60% thought they could attain at least a bachelor’s degree. We also compared aspirations and expectations among the three groups. There was no significant difference in educational aspirations for LBCOs, LBCBs, and NLBC, χ^2^ (10) = 7.35, *p* = 0.69. However, more NLBC than LBCOs and LBCBs believed that they could attain at least a master’s degree (30.5%, 20.2%, and 17.2% respectively, χ^2^ (10) = 22.33, *p* < 0.05).

Although participants reported high aspirations and expectations, there were significant differences between the parameters in the three groups (all *p* < 0.001). Then, we created a variable for the aspiration–expectation discrepancy by subtracting expectation scores from the aspiration scores, as used in previous research (e.g., Boxer et al., 2012). All but one (N = 605) reported aspirations greater than or equal to their expectations: 59.2% showed higher aspirations than expectations, and 40.6% showed aspirations equal to expectations. LBCOs and LBCBs were more likely to report educational aspiration–expectation discrepancies than NLBC (63.9%, 63.0%, and 48.8% respectively, χ^2^ (2) = 10.56, *p* < 0.01).

We then assessed the impact of several potential predictors of the aspiration–expectation discrepancy. A multiple logistic regression model was developed with aspiration–expectation discrepancy as the outcome variable (the outcome was dichotomised into two categories: students with and without a discrepancy). The full model containing all predictors (see Table 4) produced a nonsignificant Hosmer–Lemeshow statistic (χ^2^ = 5.07, *df* = 8, *p* = 0.750) and Cox and Snell’s R^2^ = 0.118 and Nagelkerke’s R^2^ = 0.160, indicating that the model could distinguish between respondents who reported and who did not report a discrepancy. Specifically, LBCOs and LBCBs were more likely to have aspirations that exceeded their expectations than NLBC. Students with better academic performance were less likely to report a discrepancy. Higher levels of mothers’ educational aspirations for children and friends’ aspirations were associated with a discrepancy, but fathers’ aspirations were not. No significant association was found between the aspiration–expectation discrepancies and the family economic status and parents’ education levels.

Lastly, Table 5 presents the comparison between adolescents with and without the educational aspiration–expectation discrepancies with regard to mental health and academic outcomes. Academic performance was used as a covariate to control its influence on the outcomes. After adjustment for academic performance, students whose aspirations were not equivalent to their expectations reported significantly lower academic self-perceptions, poorer self-regulation, and lower self-esteem than those without a discrepancy across all three groups. As to depression, there are marginally significant differences [52] between the two groups (*F* = 3.78, *p* = 0.52), adolescents with the discrepancies were more likely to report higher depression. The interaction effects were not significant (all *p* > 0.05), which indicates that there is no significant difference in the impact of educational aspiration–expectation discrepancies on mental health and academic outcomes for LBCOs, LBCBs, and NLBC. Moreover, when a cutoff score of 25 was adopted, the low-esteem risk rate of adolescents with discrepancy was significantly higher than that of adolescents with congruent aspirations and expectations (21.9% and 9.7% respectively, χ^2^ (1) = 10.75, *p* < 0.001). There are significant differences in depression risks among adolescents at the cutoff value of 4 (adolescents with the discrepancies, 43.7%, adolescents without the discrepancies, 26.7%, χ^2^ (1) = 3.86, *p* < 0.05).

## 4. Discussion

While there is a substantial body of literature investigating educational aspirations and expectations of adolescents in Western contexts, this study is the first to explore educational aspiration–expectation discrepancies among middle school students in rural China. The main findings revealed the high prevalence of aspiration–expectation discrepancies among rural adolescents, some important factor related to the discrepancies, and associated psychological and academic outcomes.

The comparisons among LBCOs, LBCBs, and NLBC in aspiration–expectation discrepancies show that there are significant differences among three groups: LBCOs and LBCBs were more likely than NLBC to believe that they were unlikely to achieve desired educational goals. Parental migration is one of the most important factors associated with discrepancies. Contrary to theories related to social remittance, our results suggest that parental migration does not enhance educational aspirations of LBC. This may be explained by previous research that correlates the impact of remittance with the economic resources of the area. Ye et al. [53] found that the positive effect on children’s educational aspirations from the migration remittance is more marked in western areas with low economic development level, compared to eastern and central areas. Our results confirm that both LBC and NLBC in Songzi county, Hubei (central China) have the same high educational aspirations, that is to go to university. This finding is related to Chinese culture and the rural context. Rural students are taught from a young age that ‘the main hope of social mobility is through education’ [54]. The emphasis on studying hard within this culture influences the attitudes held by adolescents, regardless of parental migrant status.

However, as Knight et al. [55] suggested, expectations are determined by the perceived realities faced by an individual, usually considering external constraints. In the Chinese context, due to parental absence, LBC tend to fare worse in terms of academic outcomes, such as poorer achievement [56] and lower academic satisfaction [15]. Moreover, LBC may perceive less social support from parents than NLBC [12]. These disadvantages encountered by LBC may lead to lower expectations and subsequently aspiration–expectation discrepancies. In addition, the impact of parental migration on children’s educational aspirations and expectations is shaped by various factors, such as societies’ economic status [57], children’s own gender and the gender of migrating parents [58], and cultural and social capital [14]. The associations between parental migration and educational aspirations and expectation are worthy of further exploration.

Our findings also show that the academic performance is a key predictor in explaining educational aspiration–expectation discrepancies, which is consistent with previous studies [33] and indicates that students with poorer academic performance are more likely to aspire high while expecting low. It is believed that better academic performance can enhance self-concept of students [59]. Thus, high achieving students are more likely to perceive that their hoped-for education is feasible.

Furthermore, the results of the present study add to a growing body of research on family factors associated with discrepancies. These include high educational aspirations in mothers (but not fathers). There are possible explanations. First, Chinese culture places high value on children’s obedience to and respect for their parents [60], and many parents in rural areas place a high value on education [6]. These results suggest that the aspirations of Chinese rural students are largely related to the aspirations that the parents hold for them (i.e., high mothers’ aspirations may encourage children to have such high aspirations). But the educational expectations are more influenced by external factors or realities. When these students hold high aspirations but face constraints, they are more likely to report aspiration–expectation discrepancies. Second, the differences in influences of maternal and paternal educational aspirations on discrepancies of adolescents are aligned with the cultural expectations of mother and father roles in the Chinese context. In traditional Chinese families, mothers are more involved in children’s education than fathers, and discuss educational goals with children [61,62]. In particular, the current study sample included 27.9% LBCOs, who were primarily cared for by mothers. Compared to fathers, Chinese mothers of LBC are more caring and more efficacious in promoting the educational outcomes of their children [63]. Many LBCOs living in mother-headed households may lack paternal support for education due to infrequent contact or no contact with fathers [64].

The finding of the significant impact of close friends’ aspirations on students’ aspiration–expectation discrepancies was consistent with those of some previous studies, which indicates that peer groups play an important role in adolescents’ educational goals [65,66]. Youth tend to mirror their close friends’ decisions concerning future educational trajectories, especially for those who are uncertain of their own pathways [65]. Nevertheless, since expectations are more “probabilistic assessments” [67] (p. 946), mere positive thinking is not sufficient to overcome realistic barriers to success and can cause incongruence between adolescents’ idealistic and realistic beliefs regarding educational future.

Family SES (i.e., family economic status and parents’ education levels) was not associated with discrepancies, which conflicts with previous research [33] which indicates that economically disadvantaged adolescents are more likely to have educational aspirations that exceed their expectations as they perceive more barriers. However, our results are consistent with previous research [26], which demonstrated that family SES was not related to rural students’ educational outcomes and choices. In addition, the measure of family SES in the present study used a combination of indicators, which is commonly used in research from the US and the UK. Sirin [68] suggested that the traditional measure of family SES is not as strongly associated with academic outcomes for rural adolescents, and its use in developing countries is problematic especially when measured with tools used in Western countries. One speculation for our results is that the measure may not be appropriate for our samples.

In accordance with previous research [4,33], our findings suggest that both LBC and NLBC with aspiration–expectation discrepancies reported depression risks. The result is supported by the self-discrepancy theory [28]. Discrepancy between ideal and actual selves is an important predictor of depression and anxiety [69]. A similar finding was demonstrated by Greenaway et al. [33], who indicated that the discrepant mindset (i.e., aspiring high while expecting low) is associated with depression of college students cross-sectionally and longitudinally.

Self-esteem, which is a subjective evaluation of personal value, was higher in adolescents without aspiration–expectation discrepancies. This phenomenon has been observed in setting career goals. A study of Australian high-school students showed that youth who hold different occupational aspirations to expectations reported lower self-esteem than their non-discrepancy peers [70]. These findings suggest that adolescent self-esteem is related to the perceived distance between ideal and real selves. A mechanism for the association between possible selves and self-esteem has been investigated. It has been found that matched possible selves promote goal-setting and positive thoughts about the future [71]. Having a goal can enhance motivation and self-regulatory behaviours that lead to self-improvement [72], which leads individuals to develop a positive view of themselves. In China, academic outcome is a particularly important determinant of students’ evaluations of their own worth. Thus, those with educational aspiration–expectation discrepancies are more likely to be unsatisfied with themselves.

With regard to academic outcomes, the results show that both rural LBC and NLBC with discrepancies reported poor academic self-regulating behaviours, which is consistent with previous findings in the possible selves literature, which suggests that self-regulatory behaviours are particularly important for maintaining and attaining aspirations [73,74]. This result is consistent with a previous study by Oyserman et al. [75], who also found that the mindset of aspiring high but expecting low could undermine self-regulatory effort in the academic domain. In addition, our findings were consistent with previous research indicating that adolescents with educational aspiration–expectation discrepancies tend to have lower academic self-perception [4]. This finding is supported by previous findings in the academic self-perception literature, which proposed that high academic self-perception students often set high and reachable goals, whereas high and impossible goals are more often set by those students identified as having low self-perception [76].

However, because the current study was based on cross-sectional data, it is not possible to determine the directionality and causality of the association between aspiration–expectation discrepancies and mental health and academic outcomes. The discrepancy may lead to poorer outcomes, but the converse is also possible. For example, previous research has demonstrated the reciprocal impact of academic self-perception on educational outcomes [77]. Consequently, future longitudinal work is required to examine the directionality of the associations in more detail.

## 5. Limitations and Future Research

Our study has several limitations. First, all the measures were based on self-reports, which may create the problem of common method variance and social desirability bias [78]. Second, the present study was cross-sectional, which limits causality inferences. Future research should utilise longitudinal designs to better examine the associations between the adolescents’ educational aspiration–expectation discrepancies and their academic and mental health outcomes. Third, it is possible that the measure of SES is problematic for Chinese rural students. The use of alternative measurements may be desirable. Despite these limitations, given the lack of literature on the educational aspirational aspiration–expectation discrepancy of Chinese rural LBC and NLBC, the present study has contributed to research on educational issues and well-being of rural adolescents in China. Moreover, the Chinese government has expressed increasing concern about the development and future of rural adolescents in recent years. Such findings contribute to the discourse by looking at beliefs regarding future plans and may inform future research exploring the reasons to why rural adolescents have such aspirations and expectations.

## 6. Conclusions and Implications

This is the first study to examine the educational aspiration–expectation discrepancies in Chinese rural LBC and NLBC. Parental migration was found to reduce the educational expectations of adolescents, with both LBCOs and LBCBs were more likely to have incongruent aspirations and expectations than NLBC. Other significant factors that were associated with discrepancies of the students were academic performance, mother’s educational aspirations for children, and close friends’ aspirations. Moreover, we found that both LBC and NLBC with aspiration–expectation discrepancies had poorer academic and mental health outcomes, including lower academic perception, fewer self-regulating behaviours, lower self-esteem, and higher levels of depression. Understanding factors related to aspiration–expectation discrepancies and the adverse effects of the discrepancies may provide implications for intervention programs for supporting rural adolescents, especially rural LBC.

The results of the current study have several implications for families and schools. First, parental migration is a risk factor for poorer educational outcomes. In response, the Chinese government has in recent years delivered a set of policy reforms to protect LBC welfare. For example, school-based and community-based support are of great importance for improving child development. There is encouragement to develop intervention programs that help LBC improve their coping skills with separation from parents [79]. Second, adolescents may hold lower expectations for themselves while holding high aspirations which are believed by their mothers, without considering the actual academic performance of their children [80]. Such high aspirations are not always good for students, especially for low-achieving students. From the school level, teachers could strengthen communication with parents by means of telephone and home visits such that parents are more aware about children’s learning in school. Therefore, parents may form appropriate aspirations for their children. Third, because educational aspiration–expectation discrepancies are associated with poorer academic and mental health outcomes, it is not ideal for adolescents to aspire high while simultaneously expecting low. Students’ expectations need to be raised to match aspirations more closely. As Boxer et al. [33] suggested, schools could consider designing educational initiatives to increase rural adolescents’ awareness of social and school supports that make educational goals more feasible.

## Figures and Tables

**Table 1:** Demographic information of the sample.

Variables	LBCBs (*n* = 273)	LBCOs (*n* = 169)	NLBC (*n* = 164)	*p*
	*n* (*%*)/Mean (SD)	*n* (*%*)/Mean (SD)	*n* (*%*)/Mean (SD)	
Age	14.82 (0.59)	14.87 (0.56)	14.86 (0.62)	0.65
*Sex*				0.63
Males	138 (50.5)	88 (52.1)	77 (47)	
Females	135 (49.5)	81 (47.9)	87 (53)	
*Only child*				0.86
Yes	154 (56.4)	91 (53.8)	92 (56.1)	
No	119 (43.6)	78 (46.2)	72 (43.9)	
*Parents’ marital status*				<0.001
Married	207 (75.8)	157 (92.9)	156 (95.1)	
Divorced	66 (24.2)	12 (7.1)	8 (4.9)	
*Mother’s educational level*				0.80
Primary school/below	46 (16.9)	36 (21.3)	27 (16.5)	
Middle school/Vocational school	146 (53.5)	95 (56.2)	90 (54.9)	
High school/above	40 (14.7)	22 (13.0)	26 (15.9)	
*Father’s educational level*				0.49
Primary school/below	35 (12.8)	26 (15.4)	25 (15.2)	
Middle school/Vocational school	152 (55.7)	90 (53.3)	79 (48.2)	
High school/above	48 (17.6)	32 (18.9)	38 (23.2)	
*Economic status*				0.02
Low	126 (46.2)	87 (51.5)	59 (36.0)	
Middle	114 (41.8)	57 (33.7)	72 (43.9)	
High	33 (12.1)	25 (14.8)	33 (20.1)	
**Left-behind characteristics**				
*Primary caregiver*				<0.001
Non-migrant father	-	26 (15.4)	-	
Non-migrant mother	-	143 (84.6)	-	
Grandparent(s)	266 (97.4)	-	-	
Others ^a^	7 (2.6)	-	-	
*Length of maternal migration*				<0.001
No migration	-	33 (19.5)	-	
Short-term (<1 year)	0	0	-	
Medium-term (1–5 years)	9 (3.3)	4 (2.4)	-	
Long-term (>5 years)	264 (96.7)	132 (78.1)	-	
*Length of paternal migration*				<0.001
No migration	-	7 (4.1)	-	
Short-term (<1 year)	0	0	-	
Medium-term (1–5 years)	7 (2.6)	2 (1.2)	-	
Long-term (>5 years)	266 (97.4)	160 (94.7)	-	
*Frequency of contact ^b^*				0.04
Rarely	25 (9.2)	17 (10.1)	-	
Sometimes	40 (14.7)	38 (22.5)	-	
Often	183 (67.0)	91 (53.8)	-	
Very often	25 (9.2)	23 (13.6)	-	

Note. LBCBs, children who were left behind by both parents; LBCOs, children who were left behind by one parent; NLBC, children living with both parents. ^a^ Others included 6 children living with relatives and 1 child living alone. ^b^ Frequency of phone contact with migrant parent(s): Rarely = fewer than once per month; Sometimes = at least once per month; Often = at least once per week; Very often = every day.

**Table 2:** Correlations among main variables.

	1	2	3	4	5	6	7	8	9	10	11	12
1. Aspirations	--											
2. Expectations	0.58 **	--										
3. Academic performance	0.52 **	0.74 **	--									
4. Mother’s educational aspirations	0.60 **	0.40 **	0.35 **	--								
5. Father’s educational aspirations	0.58 **	0.41 **	0.35 **	0.77 **	--							
6. Friends’ aspirations	0.51 **	0.37 **	0.26 **	0.42 **	0.42 **	--						
7. Mother’s educational level	0.06	0.13 **	0.07	0.08	0.06	0.04	--					
8. Father’s educational level	0.03	0.14 **	0.02	0.02	0.07	0.02	0.44 **	--				
9. Self-perception	0.26 **	0.44 **	0.46 **	0.18 **	0.18 **	0.12 **	0.12 **	0.07	--			
10. Self-regulation	0.29 **	0.50 **	0.44 **	0.16 **	0.19 **	0.18 **	0.05	0.09 *	0.72 **	--		
11. Depression	−0.12 **	−0.20 **	−0.18 **	−0.05	−0.07	−0.06	−0.07	−0.05	−0.43 **	−0.32 **	--	
12. Self-esteem	0.20 **	0.26 **	0.22 **	0.12 **	0.14 **	0.08 *	0.11 *	0.08	0.53 **	0.37 **	−0.71 **	--

Note. * *p* < 0.05; ** *p* < 0.01.

**Table 3:** Descriptive statistics of educational aspirations and expectations by child category.

	NLBC (*n* = 164)		LBCOs (*n* = 169)		LBCBs (*n* = 273)	
	EA *n* (%)	EE *n* (%)	EA *n* (%)	EE *n* (%)	EA *n* (%)	EE *n* (%)
1 Completion of middle school	0	1 (0.6)	1 (0.6)	4 (2.4)	0	8 (2.9)
2 Vocational school ^a^	2 (1.2)	12 (7.3)	1 (0.6)	24 (14.2)	5 (1.8)	49 (17.9)
3 High school	9 (5.5)	23 (14)	13 (7.7)	28 (16.6)	17 (6.2)	34 (12.5)
4 Bachelor’s degree	67 (40.9)	78 (47.6)	66 (39.1)	79 (46.7)	112 (41.0)	135 (49.5)
5 Master’s degree	50 (30.5)	40 (24.4)	41 (24.3)	28 (16.6)	77 (28.2)	36 (13.2)
6 A Doctoral degree	36 (22.0)	10 (6.1)	47 (27.8)	6 (3.6)	62 (22.7)	11 (4.0)
Wilcoxon signed rank test	Z = −8.12	*p* < 0.001	Z = −9.29	*p* < 0.001	Z = −11.72	*p* < 0.001

Note. NLBC, non-left-behind children; LBCOs, children who were left behind by one parent; LBCBs, children who were left behind by both parents; EA, educational aspirations; EE, educational expectations. ^a^ In China, vocational schools are not comparable to regular high schools; the former track usually focuses on vocational skills, whereas the latter track involves university preparation. Students who cannot attend regular high school are enrolled in vocational school (39.5% in 2019) [50]. Generally, regular high school students are more likely than their counterparts who attend a vocational school to move on to higher education [51].

**Table 4:** Multiple logistic regression analysis of aspiration–expectation discrepancy classification.

Predictors	*B*	SE (B)	Wald	*p*	OR	95% CI for OR
*Child category*			6.74	0.03		
NLBC					1.00	-
LBCOs	0.66	0.26	6.43	0.01	1.93	1.16–3.21
LBCBs	0.43	0.24	3.27	0.07	1.54	0.97–2.44
Sex	−0.08	0.20	0.16	0.69	0.92	0.62–1.37
Age	−0.04	0.17	0.05	0.83	0.96	0.70–1.34
Only child	−0.13	0.20	0.41	0.52	0.88	0.60–1.30
Academic performance	−0.47	0.09	28.95	0.00	0.62	0.53–0.74
*Family economic status*			0.13	0.94		
Low					1.00	-
Middle	0.04	0.22	0.03	0.86	1.04	0.68–1.59
High	−0.06	0.29	0.05	0.83	0.94	0.53–1.67
Mother’s education	−0.16	0.10	2.35	0.13	0.85	0.70–1.05
Father’s education	−0.12	0.10	1.50	0.22	0.89	0.73–1.08
Mother’s aspirations	0.41	0.18	5.25	0.02	1.50	1.06–2.13
Father’s aspirations	0.12	0.18	0.42	0.52	1.12	0.79–1.60
Friends’ aspirations	0.27	0.12	5.34	0.02	1.31	1.04–1.64
Constant	−0.63	2.68	0.06	0.81	0.53	

Note. *B*, regression coefficient, OR, odds ratio, CI, confidence interval.

**Table 5:** Differences in academic outcomes and well-being by aspiration–expectation discrepancy status and child category.

	LBCBs (*n* = 273)	LBCOs (*n* = 169)	NLBC (*n* = 164)	Discrepancy (*n* = 360)	No Discrepancy (*n* = 246)	Child Category	Discrepancy	Interaction
	Mean (*SD*)	Mean (*SD*)	Mean (*SD*)	Mean (*SD*)	Mean (*SD*)	*F*	*F*	*F*
Academic self-perception	26.42 (8.25)	26.73 (8.02)	28.34 (8.72)	25.74 (8.21)	28.91 (8.2)	0.26	12.49 ***	1.29
Academic self-regulation	47.02 (11.62)	47.44 (12.25)	49.11 (10.97)	46.29 (11.91)	49.77 (10.95)	0.06	5.90 *	0.63
Depression	6.05 (3.82)	6.31 (3.93)	5.52 (3.47)	6.33 (3.79)	5.48 (3.67)	0.81	3.78	0.07
Self-esteem	26.61 (6.10)	26.72 (6.06)	27.48 (5.62)	26.25 (6.1)	27.78 (5.65)	0.18	4.69 *	0.49

Note. * *p* < 0.05; ** *p* < 0.01; *** *p* < 0.001.

## References

[1] Khattab N. (2015). Students’ aspirations, expectations and school achievement: What really matters?. Br. Educ. Res. J..

[2] Sharp E.H., Seaman J., Tucker C.J., Van Gundy K.T., Rebellon C.J. (2020). Adolescents’ future aspirations and expectations in the context of a shifting rural economy. J. Youth Adolesc..

[3] Massey E.K., Gebhardt W.A., Garnefski N. (2008). Adolescent goal content and pursuit: A review of the literature from the past 16 years. Dev. Rev..

[4] Kirk C.M., Lewis R.K., Scott A., Wren D., Nilsen C., Colvin D.Q. (2012). Exploring the educational aspirations–expectations gap in eighth grade students: Implications for educational interventions and school reform. Educ. Stud..

[5] Archer L., Francis B. (2006). Challenging classes? Exploring the role of social class within the identities and achievement of British Chinese pupils. Sociology.

[6] Koo A. (2012). Is there any chance to get ahead? Education aspirations and expectations of migrant families in China. Br. J. Sociol. Educ..

[7] Wu X., Treiman D.J. (2007). Inequality and equality under Chinese socialism: The hukou system and intergenerational occupational mobility. Am. J. Sociol..

[8] Postiglione G.A., Xie A.L., Jung J.S., Hong Y.B. (2017). Rural students in Chinese top-tier university: Family background, school effects, and academic performance. Chin. Educ. Soc..

[9] UNICEF China (2017). Population Status of Children in China in 2015: Facts and Figures. https://www.unicef.cn.

[10] Li H., Loyalka P., Rozelle S., Wu B., Xie J. (2015). Unequal access to college in China: How far have poor, rural students been left behind?. China Q..

[11] National Bureau of Statistics (2020). Investigation Report on Migrant Workers in 2019. http://www.stats.gov.cn/tjsj/zxfb/202004/t20200430_1742724.html.

[12] Sun X., Tian Y., Zhang Y., Xie X., Heath M.A., Zhou Z. (2015). Psychological development and educational problems of left-behind children in rural China. Sch. Psychol. Int..

[13] Duan C., Zhou F. (2005). Research on the left-behind children in China. J. Popul. Res..

[14] Liang Z., Song Q. (2018). From the culture of migration to the culture of remittances: Evidence from immigrant-sending communities in China. Chin. Sociol. Rev..

[15] Wen M., Lin D. (2012). Child development in rural China: Children left behind by their migrant parents and children of nonmigrant families. Child Dev..

[16] Lahaie C., Hayes J.A., Piper T.M., Heymann J. (2009). Work and family divided across borders: The impact of parental migration on Mexican children in transnational families. Community Work Fam..

[17] Hu F. (2013). Does migration benefit the schooling of children left behind? Evidence from rural northwest China. Demogr. Res..

[18] He B., Fan J., Liu N., Li H., Wang Y., Williams J., Wong K. (2012). Depression risk of ‘left-behind children’ in rural China. Psychiatry Res..

[19] Fan F., Su L., Gill M.K., Birmaher B. (2010). Emotional and behavioral problems of Chinese left-behind children: A preliminary study. Soc. Psychiatry Psychiatr. Epidemiol..

[20] McKenzie D., Rapoport H. (2011). Can migration reduce educational attainment? Evidence from Mexico. J. Popul. Econ..

[21] Ye J.Z., Pan L. (2011). Differentiated childhoods: Impacts of rural labor migration on left-behind children in China. J. Peasant Stud..

[22] Spera C., Wentzel K.R., Matto H.C. (2009). Parental aspirations for their children’s educational attainment: Relations to ethnicity, parental education, children’s academic performance, and parental perceptions of school climate. J. Youth Adolesc..

[23] Agger C., Meece J., Byun S.-Y. (2018). The influences of family and place on rural adolescents’ educational aspirations and post-secondary enrollment. J. Youth Adolesc..

[24] Bronstein P., Ginsburg G.S., Herrera I.S. (2005). Parental predictors of motivational orientation in early adolescence: A longitudinal study. J. Youth Adolesc..

[25] Hesketh T., Ding Q.J. (2005). Anxiety and depression in adolescents in urban and rural China. Psychol. Rep..

[26] Zhang Y. (2016). The Hopes Carry Them On: Early Educational Expectations and Later Educational Outcomes in Rural Gansu, China. Fam. Environ. Sch. Resour. Educ. Outcomes.

[27] Yiu L., Yun L. (2017). China’s rural education: Chinese migrant children and left-behind children. Chin. Educ. Soc..

[28] Higgins E.T. (1987). Self-discrepancy: A theory relating self and affect. Psychol. Rev..

[29] Liw L., Han S.Y. (2020). Coping as a moderator of self-discrepancies and psychological distress. Couns. Psychol. Q..

[30] Gürcan-Yıldırım D., Gençöz T. (2020). The association of self-discrepancy with depression and anxiety: Moderator roles of emotion regulation and resilience. Curr. Psychol..

[31] Rutherford T. (2015). Emotional well-being and discrepancies between child and parent educational expectations and aspirations in middle and high school. Int. J. Adolesc. Youth.

[32] Greenaway K.H., Frye M., Cruwys T. (2015). When aspirations exceed expectations: Quixotic hope increases depression among students. PLoS ONE.

[33] Boxer P., Goldstein S.E., DeLorenzo T., Savoy S., Mercado I. (2011). Educational aspiration–expectation discrepancies: Relation to socioeconomic and academic risk-related factors. J. Adolesc..

[34] Freitas A.L., Higgins E.T. (2002). Enjoying goal-directed action: The role of regulatory fit. Psychol. Sci..

[35] Oyserman D., Fryberg S.A., Yoder N. (2007). Identity-based motivation and health. J. Personal. Soc. Psychol..

[36] Oyserman D., James L., Markman K.D., Klein W.M.P., Suhr J.A. (2009). Possible selves: From content to process. Handbook of Imagination and Mental Simulation.

[37] Leung H., Wu F.K.Y., Shek D.T.L. (2017). Hope, aspirations, and resilience in children and adolescents: A review of research on measurement and related antecedents. Int. J. Disabil. Hum. Dev..

[38] Zhao C., Wang F., Li L., Zhou X., Hesketh T. (2017). Long-term impacts of parental migration on Chinese children’s psychosocial well-being: Mitigating and exacerbating factors. Soc. Psychiatry Psychiatr. Epidemiol..

[39] Currie C.E., Elton R.A., Todd J., Platt S. (1997). Indicators of socioeconomic status for adolescents: The WHO Health Behaviour in School-aged Children Survey. Health Educ. Res..

[40] Liu Y., Wang M., Villberg J., Torsheim T., Tynjälä J., Lv Y., Kannas L. (2012). Reliability and validity of Family Affluence Scale (FAS II) among adolescents in Beijing, China. Child Indic. Res..

[41] Wu N., Hou Y., Wang Q., Yu C. (2018). Intergenerational transmission of educational aspirations in Chinese families: Identifying mediators and moderators. J. Youth Adolesc..

[42] McCoach D.B., Siegle D. (2003). The school attitude assessment survey-revised: A new instrument to identify academically able students who underachieve. Educ. Psychol. Meas..

[43] Zimmerman B.J., Schunk D.H. (2001). Self-Regulated Learning and Academic Achievement: Theoretical Perspectives.

[44] Kovacs M. (1992). Children’s Depression Inventory: Manual.

[45] Sun S., Wang S. (2015). The Children’s Depression Inventory in worldwide child development research: A reliability generalization study. J. Child Fam. Stud..

[46] Allgaier A.K., Frühe B., Pietsch K., Saravo B., Baethmann M., Schulte-Körne G. (2012). Is the Children’s Depression Inventory Short version a valid screening tool in pediatric care? A comparison to its full-length version. J. Psychosom. Res..

[47] Rosenberg M. (1965). Society and the Adolescent Self-Image.

[48] Isomaa R., Väänänen J.M., Fröjd S., Kaltiala-Heino R., Marttunen M. (2013). How low is low? Low self-esteem as an indicator of internalizing psychopathology in adolescence. Health Educ. Behav..

[49] Wu Y., Zuo B., Wen F.F., Yan L. (2017). Rosenberg self-esteem scale: Method effects, factorial structure and scale invariance across migrant child and urban child populations in China. J. Personal. Assess..

[50] Ministry of Education of the People’s Republic of China (2020). Composition of Students in Senior Secondary Schools.

[51] Chen D., Fu N., Pan Y. (2019). Progress and Challenges of Upper Secondary Education in China.

[52] Lakens D. (2015). On the challenges of drawing conclusions from *p*-values just below 0.05. PeerJ.

[53] Ye J.Y., Zhang R., Wang Q. (2017). Parental migration and the educational aspiration for left-behind children—An empirical analysis based on 2010 CFPS data. Econ. Sci..

[54] Zhang D., Li X., Xue J. (2015). Education inequality between rural and urban areas of the People’s Republic of China, migrants’ children education, and some implications. Asian Dev. Rev..

[55] Knight K.E., Ellis C., Roark J., Henry K.L., Huizinga D. (2017). Testing the role of aspirations, future expectations, and strain on the development of problem behaviors across young and middle adulthood. Deviant Behav..

[56] Song S., Chen C., Zhang A. (2018). Effects of parental migration on life satisfaction and academic achievement of left-behind children in rural China—A case study in Hubei province. Children.

[57] Sun F., Liu Z., Schiller K.S. (2020). Parental migration and children’s educational aspirations: China and Mexico in a comparative perspective. Chin. Sociol. Rev..

[58] Lu Y. (2014). Parental migration and education of left-behind children: A comparison of two settings. J. Marriage Fam..

[59] Strayhorn T.L. (2009). Different folks, different hopes: The educational aspirations of Black males in urban, suburban, and rural high schools. Urban Educ..

[60] Ho D.Y.F., Spinks J.A., Yeung C.S.H. (1989). Chinese Patterns of Behavior: A Sourcebook of Psychological and Psychiatric Studies.

[61] Tam V.C. (2009). A comparison of fathers’ and mothers’ contributions in the prediction of academic performance of school-age children in Hong Kong. Int. J. Psychol..

[62] Shek D.T. (2005). Perceived parental control and parent–child relational qualities in Chinese adolescents in Hong Kong. Sex Roles.

[63] Zhang Y., Kao G., Hannum E. (2007). Do mothers in rural China practice gender equality in educational aspirations for their children?. Comp. Educ. Rev..

[64] Jia Z., Tian W. (2010). Loneliness of left-behind children: A cross-sectional survey in a sample of rural China. Child Care Health Dev..

[65] Kiuru N., Aunola K., Vuori J., Nurmi J.E. (2007). The role of peer groups in adolescents’ educational expectations and adjustment. J. Youth Adolesc..

[66] Hemi A., Madjar N., Rich Y. (2021). Perceived peer and teacher goals: Relationships with students’ academic achievement goals. J. Exp. Educ..

[67] Baird C.L., Burge S.W., Reynolds J.R. (2008). Absurdly ambitious? Teenagers’ expectations for the future and the realities of social structure. Sociol. Compass.

[68] Sirin S.R. (2005). Socioeconomic status and academic achievement: A meta-analytic review of research. Rev. Educ. Res..

[69] Mason T.B., Smith K.E., Engwall A., Lass A., Mead M., Sorby M., Bjorlie K., Strauman T.J., Wonderlich S. (2019). Self-discrepancy theory as a transdiagnostic framework: A meta-analysis of self-discrepancy and psychopathology. Psychol. Bull..

[70] Patton W., Creed P. (2007). Occupational aspirations and expectations of Australian adolescents. Aust. J. Career Dev..

[71] Hoyle R.H., Sherrill M.R. (2006). Future orientation in the self-system: Possible selves, self-regulation, and behavior. J. Personal..

[72] King L.A. (2001). The health benefits of writing about life goals. Personal. Soc. Psychol. Bull..

[73] Lee S.J., Oyserman D. (2007). Reaching for the future: The education-focused possible selves of low-income mothers. New Dir. Teach. Learn..

[74] Oyserman D., Bybee D., Terry K., Hart-Johnson T. (2004). Possible selves as roadmaps. J. Res. Personal..

[75] Oyserman D., Destin M., Novin S. (2015). The context-sensitive future self: Possible selves motivate in context, not otherwise. Self Identity.

[76] Hamachek D. (1995). Self-Concept and School Achievement: Interaction Dynamics and a Tool for Assessing the Self-Concept Component. J. Couns. Dev..

[77] Uwah C.J., McMahon H.G., Furlow C.F. (2008). School belonging, educational aspirations, and academic self-efficacy among African American male high school students: Implications for school counselors. Prof. Sch. Couns..

[78] Lindell M.K., Whitney D.J. (2001). Accounting for common method variance in cross-sectional research designs. J. Appl. Psychol..

[79] All-China Women’s Federation (2016). Opinions of the State Council on Strengthening the Care and Protection of Rural Left-Behind Children. www.gov.cn/zhengce/content/2016-02/14/content_5041066.htm.

[80] Brown P.H. (2006). Parental education and investment in children’s human capital in rural China. Econ. Dev. Cult. Chang..

